# Mitochondrial ATF2 translocation contributes to apoptosis induction and BRAF inhibitor resistance in melanoma through the interaction of Bim with VDAC1

**DOI:** 10.18632/oncotarget.5537

**Published:** 2015-10-09

**Authors:** Zongwei Gao, Qingjuan Shang, Zhaoyun Liu, Chun Deng, Chunbao Guo

**Affiliations:** ^1^ Laboratory of Surgery, Children's Hospital of Chongqing Medical University, Children's Hospital of Chongqing Medical University, Ministry of Education Key Laboratory of Child Development and Disorders, Chongqing, P. R. China; ^2^ Department of Pathology, Linyi People's Hospital, Linyi, Shandong, P. R. China; ^3^ Department of Neonatology, Children's Hospital of Chongqing Medical University, Chongqing, P. R. China

**Keywords:** mitochondria, ATF2, Bim, VDAC1, Mcl-1

## Abstract

**Background:**

The mitochondrial accumulation of ATF2 is involved in tumor suppressor activities via cytochrome c release in melanoma cells. However, the signaling pathways that connect mitochondrial ATF2 accumulation and cytochrome c release are not well documented.

**Methods:**

Several melanoma cell lines, B16F10, K1735M2, A375 and A375-R1, were treated with paclitaxel and vemurafenib to test the function of mitochondrial ATF2 and its connection to Bim and voltage-dependent anion channel 1 (VDAC1). Immunoprecipitation analysis was performed to investigate the functional interaction between the involved proteins. VDAC1 oligomerization was evaluated using an EGS-based crosslinking assay.

**Results:**

The expression and migration of ATF2 to the mitochondria accounted for paclitaxel stimuli and acquired resistance to BRAF inhibitors. Mitochondrial ATF2 facilitated Bim stabilization through the inhibition of its degradation by the proteasome, thereby promoting cytochrome c release and inducing apoptosis in B16F10 and A375 cells. Studies using B16F10 and A375 cells genetically modified for ATF2 indicated that mitochondrial ATF2 was able to dissociate Bim from the Mcl-1/Bim complex to trigger VDAC1 oligomerization. Immunoprecipitation analysis revealed that Bim interacts with VDAC1, and this interaction was remarkably enhanced during apoptosis.

**Conclusion:**

These results reveal that mitochondrial ATF2 is associated with the induction of apoptosis and BRAF inhibitor resistance through Bim activation, which might suggest potential novel therapies for the targeted induction of apoptosis in melanoma therapy.

## INTRODUCTION

Among human malignancies, melanoma is extremely aggressive and resistant to treatment [1], and the incidence of melanoma continues to increase worldwide. BRAF mutations play a critical role in melanoma oncogenesis and have thus been shown to be a promising target for molecular therapeutic approaches. Vemurafenib, the first highly selective BRAFV600E inhibitor, elicited unprecedented success for both response rate and overall survival [2, 3]. However, almost all of the patients eventually developed drug resistance and relapsed within 6 to 8 months [3]. A critical issue moving forward is to address the molecular mechanisms underlying the resistance of mutant BRAF-expressing melanoma cells to B-RAF inhibition.

Activating transcription factor 2 (ATF2), a member of the activator protein 1 (AP1) transcription factor superfamily [4, 5], can homodimerize or heterodimerize with other members of the AP1 family to regulate diverse cellular functions [6, 7]. Depending on its subcellular localization, ATF2 can elicit divergent functions of oncogenic or tumor suppressor activities [4, 8]. The nuclear localization of ATF2 coincides with poor prognosis in melanoma patients [4, 9], and the forced expression of cytoplasmic ATF2 peptides induces apoptosis of melanoma cells [10]. ATF2 mitochondrial localization is primarily regulated by PKCε, which enables its mitochondrial function. Thr52 is a PKCε phosphoacceptor site [4]. In other tumors, tumor suppressor activity of cytosolic ATF2 has also been observed [11, 12, 13]. Further studies have shown that cytoplasmic ATF2 binds to VDAC1 to regulate mitochondrial outer membrane permeabilization (MOMP), thereby controlling the release of cytochrome c and promoting apoptosis [4]. Regarding the mechanism of MOMP regulation, BH3-only proteins (BH3s), which contain only a BH3 domain, such as Bim, Puma, and Noxa regulate the activation of the pro-apoptotic proteins Bax and Bak to form a mitochondrial outer membrane channel for cytochrome c release [14, 15, 16, 17]. Resistance to BRAF inhibitors might involve the suppression of Bim-mediated apoptosis through the inhibition of Bim expression [18, 19, 20]. Bim-deficient lymphocytes are resistant to several apoptotic stimuli, including cytokine deprivation, Ca^2+^ ionophores, and Taxol [21, 22].

Evidence has shown that although the affinity of Bim for Bcl-2, Bcl-xL, and Bcl-w is high [23, 24], in response to apoptotic stimuli, Bim may be released and translocate to the mitochondria to initiate MOMP [25, 26], which controls the efflux of apoptogenic factors such as cytochrome c [27]. According to these results, we speculate that there might be a signaling relationship between the ATF2 and BH3 proteins, which are involved in mitochondria-based apoptosis and BRAF inhibitor resistance in melanoma.

Here, we investigate the role of cytosolic ATF2 in mitochondrial apoptosis and acquired BRAF inhibitor resistance and the mechanisms underlying the interaction of ATF2 with other pro-apoptotic proteins. In the present study, we characterize the BH3-only protein Bim in the apoptosis-induced increase of mitochondrial membrane permeability induced by ATF2 in the mitochondria. Our work revealed that following ATF2 localization in the mitochondria, Bim is released from Mcl-1 and then activates voltage-dependent anion channel-1 (VDAC1) for cytochrome c release. Furthermore, VDAC1 oligomerization is involved in apoptosis. These studies reveal novel insights into the mechanisms underlying the function of mitochondrial ATF2, which might provide mechanistic approaches to restore the tumor suppressor function of ATF2 and overcome resistance to BRAF inhibitors and other cytotoxic agents during melanoma treatment.

## RESULTS

### Identification of ATF2/Bim involvement in apoptosis and BRAF inhibitor resistance

**Figure 1:**
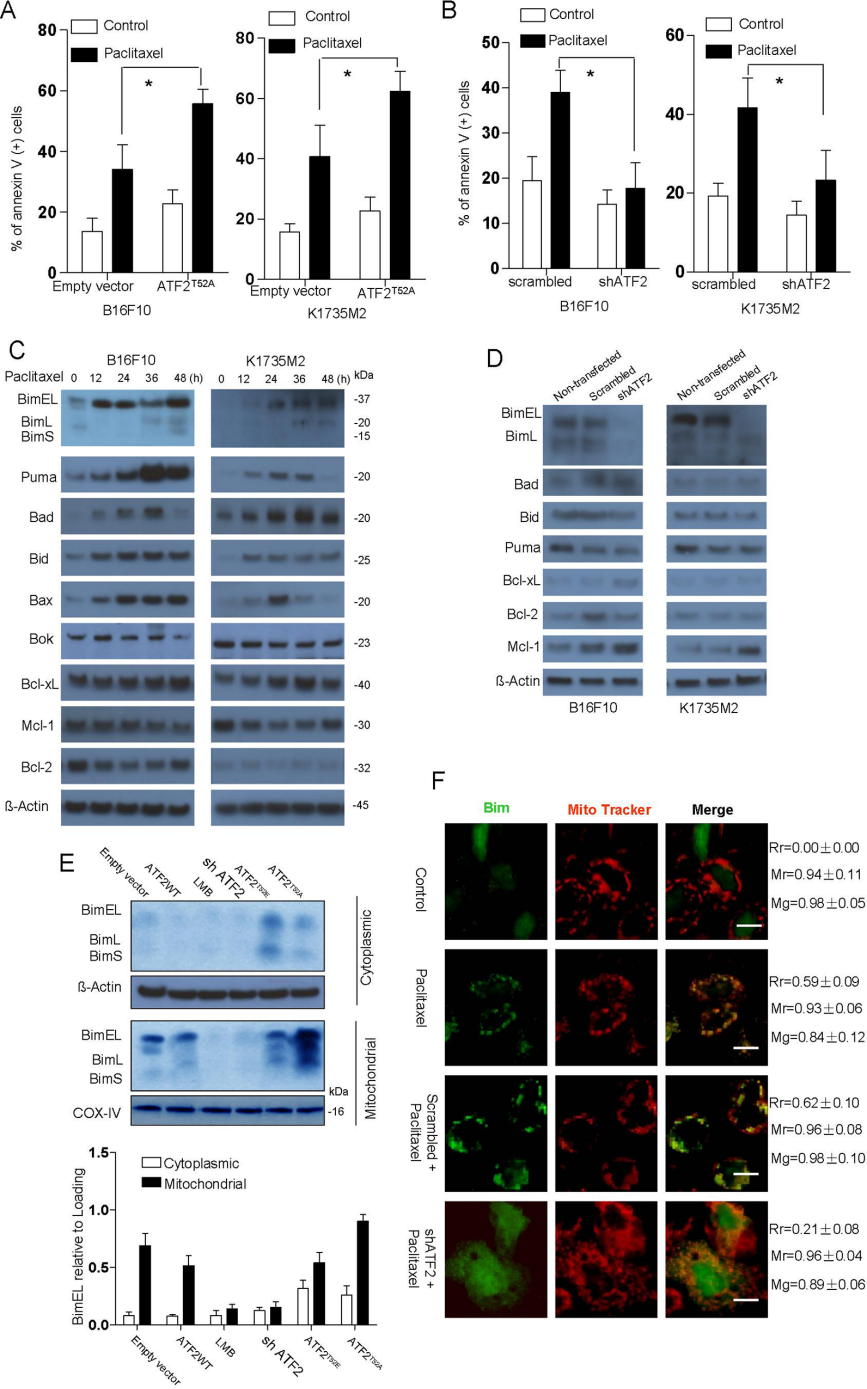
ATF2-associated apoptosis in B16F10 and K1735M2 cells is mainly dependent on Bim B16F10 cells (left panel) and K1735M2 cells (right panel) transfected with empty vector (EV) or ATF2^T52A^ **A.** and scrambled or ATF2-specific shRNA **B.** were treated with paclitaxel (100 nM) for 24 h and subjected to apoptotic cell death analysis using Annexin V/PI staining. Columns represent the average values of three independent experiments; bars, s.e.m. *, *p* < 0.01 compared with the corresponding control groups (by one-way ANOVA). **C.** Immunoblotting analysis of the indicated proteins in the whole-cell lysates of B16F10 and K1735M2 cells that were treated with paclitaxel (100 nM) for the indicated times. The data are representative of three independent experiments, and the protein weights are indicated (in kDa). **D.** Immunoblotting analysis of the indicated proteins in the whole-cell lysates of B16F10 and K1735M2 cells stably transfected with scrambled or ATF2-specific shRNA that were treated with paclitaxel (100 nM) for 24 h. Representative figures of multiple experiments are shown. **E.** B16F10 cells were transfected with empty vector (EV), ATF2 (WT), ATF2^T52A^, or ATF2^T52E^, as well as ATF2 shRNA, and then treated with paclitaxel (100 nM, 12 h). Alternatively, prior to paclitaxel treatment, the mitochondrial accumulation of ATF2 was prevented with Leptomycin B (LMB, 40 ng/ml, 6 h). Cytosolic and mitochondrial fractions were subjected to western blot analysis with antibodies against Bim. β-actin and COX-IV were probed as loading controls. Representative figures of multiple experiments are shown. Lower panel: Quantitative data of relative intensity for E were calculated with respect to the corresponding loading control (*n* = 3). **F.** Confocal microscopy images of B16F10 cells transfected with scrambled or ATF2-specific shRNA that were treated with paclitaxel and stained with a Bim-specific antibody. The data are representative of three independent replicate coverslips per condition. Scale bar: 20 μm. Rr, Pearson's correlation coefficient; Mr, Mander's co-localization coefficient for red; Mg, Mander's co-localization coefficient for green.

We first examined ATF2 localization and performed apoptosis induction assays in a panel of melanoma cell lines, including B16F10, K1735M2, A375 and A375-R1 cells. ATF2 was localized to the mitochondria by paclitaxel and vemurafenib treatment in a time-dependent manner (Supplementary Figure S1A, S1B). At the same time points, all of the tested cell types were sensitive to apoptosis induction by the same stimuli (Figure 1A, 1B). We also observed ATF2 mitochondrial translocation in A375 cells but not in the BRAF inhibitor-resistant A375R cells, which is likely responsible for vemurafenib resistance (Supplementary Figure S1B). Furthermore, consistent with previous reports that the ATF2^T52A^ mutant localized to the cytoplasm and mitochondria and the ATF2^T52E^ mutant exhibited constitutive nuclear localization, the localization of ATF2 to the mitochondria by the exogenous expression of ATF2^T52A^ sensitized B16F10 and K1735M2 cells to paclitaxel (Figure 1A) and A375-R1 cells to vemurafenib (Supplementary Figure S1E). Furthermore, ATF2 depletion by shRNA caused cells to become resistant to apoptosis induced by paclitaxel (Figure 1B), suggesting that paclitaxel-induced apoptosis and the restoration of vemurafenib sensitivity might act via the mitochondrial localization of ATF2.

We next analyzed the level of the Bcl-2 family of proteins following cytotoxic stimulation. As shown in Figure 1C, the expression of Puma, Bid, Bad, and Bax was initially enhanced (within 12 hrs) and then declined in B16F10 and K1735M2 cells, with the exception of for Bid (in both cell lines) and Bax (in B16F10 cells). There were no significant changes in Bok, Mcl-1, Bcl-2 and Bcl-xL expression up to 48 h after paclitaxel treatment. In contrast, BimEL, BimL, and BimS levels were elevated by 2- to 5-fold in cells 48 h after paclitaxel treatment. Notably, coincident with ATF2 translocation, the expression levels of Bim proteins were elevated as early as 12 h after paclitaxel treatment. Furthermore, we observed no significant change in Bad, Bid, or Bax expression but did observe a decrease in Bim levels when ATF2 was depleted in shATF2-infected B16F10 cells (Figure 1D). A slight decrease in Puma expression was observed in shATF2-infected B16F10 cells but not in K1735M2 cells. Additionally, for Mcl-1, an increase in Mcl-1 protein levels upon ATF2 down-regulation was detected. Consistent with this, the mRNA expression of Bim was rapidly (within 12 h) and robustly induced by vemurafenib in A375 cells but not in A375R cells (Supplementary Figure S2B). We inferred than Bim might be the predominant effector for cytotoxic effects and BRAF inhibitor resistance compared with the other related BH3-only proteins Bid or Puma in B16F10, K1735M2 and A375R cells.

We next addressed whether there was a causative mechanism for the concomitant induction of ATF2 and Bim. Remarkably, when ATF2 was depleted, paclitaxel-induced upregulation of Bim in B16F10 cells was potently inhibited to minimal levels, whereas the expression of the anti-apoptotic BCL-2 family member Mcl-1 was slightly increased. In contrast, Bcl-2 and Bcl-xL levels were slightly decreased upon ATF2 depletion (Figure 1D). Further subcellular fractionation showed that most of the three Bim isoforms generated by expression of ATF2^T52A^ were located in the mitochondrial fraction (Figure 1E), and immunostaining of the cells with Bim-specific antibodies confirmed the mitochondrial localization of Bim (Figure 1F). Leptomycin B (LMB), thea nuclear export inhibitor, whichthat could prevents mitochondrial accumulation of ATF2, suppressed the mitochondrial localization of Bim (Figure 1E). In the absence of ATF2, Bim was diffusely distributed in the cytosol and nuclei of B16F10 cells, which did not change after paclitaxel treatment. This result suggested that Bim expression was mainly promoted by mitochondrial ATF2 and was not related to nuclear ATF2. Notably, the expression of Bim proteins was enhanced as early as 12 h after paclitaxel treatment, which was coincident with the release of cytochrome c (Figure 1C, Supplementary Figure S1F), suggesting that Bim proteins may be critical for the ATF2-induced apoptotic mitochondrial changes that commit cells to death.

### Bim is not a direct target gene of ATF2 signaling in B16F10 and A375 cells

Upregulation of Bim expression could potentially occur at the level of transcription, translation, and/or protein stabilization [28]. To test the mechanism of ATF2-mediated regulation of Bim expression, we pursued the question of whether Bim is an immediate, early target of ATF2 signaling (by directly upregulating Bim transcription). Approximately 2-fold (B16F10 cells) and 12-fold (A375 cells) increases in Bim transcript levels were observed after paclitaxel and vemurafenib treatment (determined by qRT-PCR) (Supplementary Figure S2A, Figure S2B), whereas Bim protein levels (determined by Western blot) increased by approximately 5-fold (Figure 1C).

**Figure 2:**
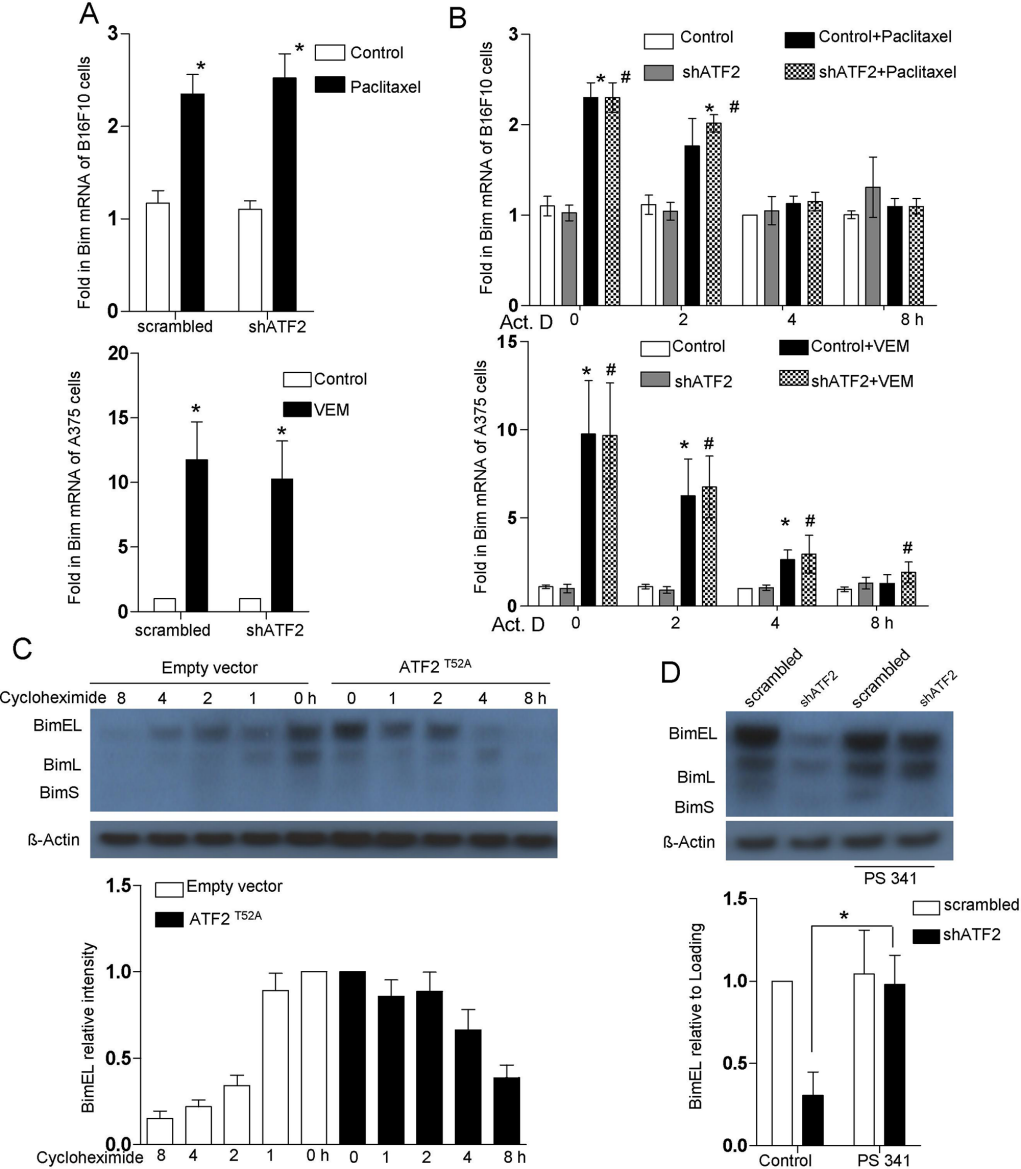
Bim protein stabilization is regulated by ATF2 **A.** Bim mRNA levels in B16F10 cells transfected with ATF2-specific shRNA in the presence or absence of paclitaxel (100 nM) for 24 h (upper panel) and in A375 cells transfected with ATF2-specific shRNA in the presence or absence of vemurafenib (5 μM) for 24 h (lower panel). RNA levels are expressed relative to the levels detected in the 2 h untreated sample (assigned *a* value of 1; bars, s.e.m.). At least three independent experiments were performed. *p < 0.01 compared with the corresponding control groups (one-way ANOVA). **B.** Bim expression analysis in B16F10 cells (upper panel) and A375 cells (lower panel) was performed by a time course treatment of the transcriptional inhibitor actinomycin D (ActD, 2.5 μg/ml), followed by paclitaxel and vemurafenib treatment for 24 h, respectively. RNA levels are expressed relative to the levels detected in the 2 h untreated sample (assigned *a* value of 1; bars, s.e.m.). At least three independent experiments were performed. *p < 0.01, #*p* < 0.01 compared with the corresponding control groups (one-way ANOVA). **C.** Immunoblot for Bim in whole-cell lysates of B16F10 cells that were stably transfected with empty vector (EV) or ATF2^T52A^ following cycloheximide (CHX, 50 μg/ml) incubation for the indicated times. The data are representative of three independent experiments. **D.** B16F10 cells transfected with ATF2-specific shRNA were treated with paclitaxel for 24 hr with or without the addition of the inhibitor PS341 (100 nM). Whole-cell lysates were subjected to western blot for Bim. Probing with antibody against β-actin was used as a loading control. Lower panel: Quantitative data of relative intensity for D and **E.** were calculated with respect to the sample at 0 h (assigned *a* value of 1).

Actinomycin D, a known transcriptional inhibitor, blocked the paclitaxel-induced increase of Bim mRNA levels, indicating that the increase in Bim transcription levels could be attributed to a transcriptional increase rather than a change in mRNA stability (Figure 2B). Importantly, ATF2 depletion had no significant effect on Bim mRNA induction in B16F10 and A375 cells (Figure 2A). These results indicated that the half-life of Bim mRNA was not altered by ATF2 depletion, suggesting that Bim was not transcriptionally upregulated by ATF2. We next investigated whether ATF2 was able to increase Bim levels through a post-translational mechanism affecting Bim protein stability. Immunoblotting analysis revealed that following cycloheximide treatment, the half-life of Bim protein was considerably elevated in ATF2^T52A^-expressing B16F10 cells (Figure 2C). The proteasome inhibitor PS 341 restored Bim protein levels in ATF2-depleted B16F10 cells (Figure 2D). These results indicate that during paclitaxel treatment, cytosolic ATF2 may inhibit proteasomal degradation, thereby leading to Bim accumulation in B16F10, K1735M2 and A375 cells.

### Bim mitochondrial localization is required for ATF2-associated apoptotic changes in the mitochondria and apoptosis

**Figure 3:**
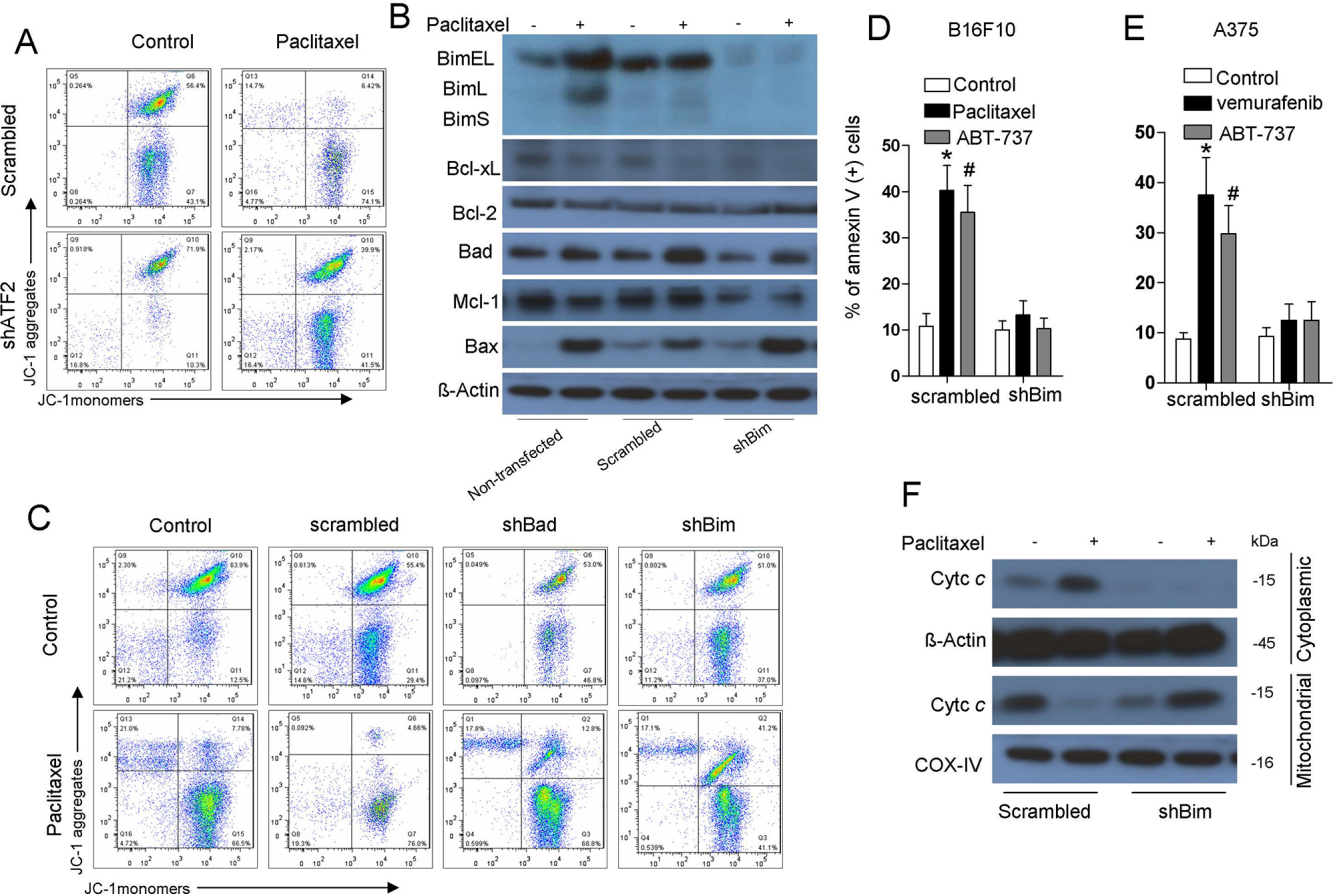
Induction of Bim is critical for ATF2-associated apoptotic changes of the mitochondria and apoptosis **A.** B16F10 cells stably transfected with ATF2-specific shRNA were treated with paclitaxel (100 nM) for 24 h. JC-1 flow cytometry analysis was performed, and representative flow cytometry plots of multiple experiments are shown. **B.** B16F10 cells stably transfected with scrambled or Bim-specific shRNA were treated with paclitaxel (100 nM) for 24 h. A western blot of the indicated proteins in whole-cell lysates is shown. β-actin was included as a loading control. **C.** B16F10 cells stably transfected with scrambled or Bim shRNA and Bad shRNA were treated with paclitaxel for 24 h. JC-1 flow cytometry analysis was performed, and representative flow cytometry plots are shown. B16F10 cells **D.** expressing scrambled or Bim shRNA were subjected to treatment with paclitaxel (100 nM) or ABT-737 (1 μM) for 24 h, and A375 cells **E.** expressing scrambled or Bim shRNA were subjected to vemurafenib (5 μM) treatment for 24 h. Apoptosis was then measured by Annexin V/PI staining (bars, s.e.m.) (*n* = 3). Columns represent the mean percentage of annexin V-positive cells from at least three independent experiments; bars, s.e.m. *p < 0.01 compared with the corresponding control groups (one-way ANOVA). **F.** B16F10 cells expressing scrambled or Bim shRNA were treated with paclitaxel (100 nM) for 24 h and subjected to western blotting for cytochrome c in the cytosolic and mitochondrial fractions. β-actin and COX-IV were used as loading controls. Representative figures of multiple experiments are shown.

We next attempted to investigate whether Bim plays an essential role in the apoptotic changes in the mitochondria induced by ATF2. Cytotoxic stress reduced mitochondrial membrane potential, as determined by alterations in JC-1 dye aggregation, whereas ATF2 depletion did not alter either of the fluorescence signals (Figure 3A). We further stably knocked down Bim expression using a commercially available shRNA specific to all Bim isoforms (Supplementary Figure S3A). This shBim did not alter the expression of other Bcl-2 family members (Figure 3B). Bim depletion (Figure 3C) eliminated alterations in the fluorescence signals of JC-1 induced by paclitaxel treatment. The induction of apoptosis (Figure 3D, 3E) and cytochrome c release (Figure 3F, Supplementary Figure S3B) were also potently inhibited by Bim depletion. Consistent with B16F10 cells, in A375 cells, depletion of Bim exhibited consistent effects on apoptosis and cytochrome c release following vemurafenib treatment (Figure 3E, Supplementary Figure S3B).

Furthermore, we used ABT-737, a Bcl-2 homology domain 3 (BH3) mimetic that is thought to act by displacing Bim from Bcl-2, to restore paclitaxel sensitivity of ATF2-depleted cells [23, 29]. As expected, introduction of ABT-737 sensitized cells to paclitaxel treatment via mitochondrial membrane potential alterations (Supplementary Figure S3C), even when ATF2 was depleted. ABT-737 also sensitized ATF2-depleted cells to paclitaxel treatment-induced cell death to the same extent as control cells (Supplementary Figure S3D).

### ATF2 induces mitochondrial VDAC upregulation and dimerization in a Bim-dependent manner

**Figure 4:**
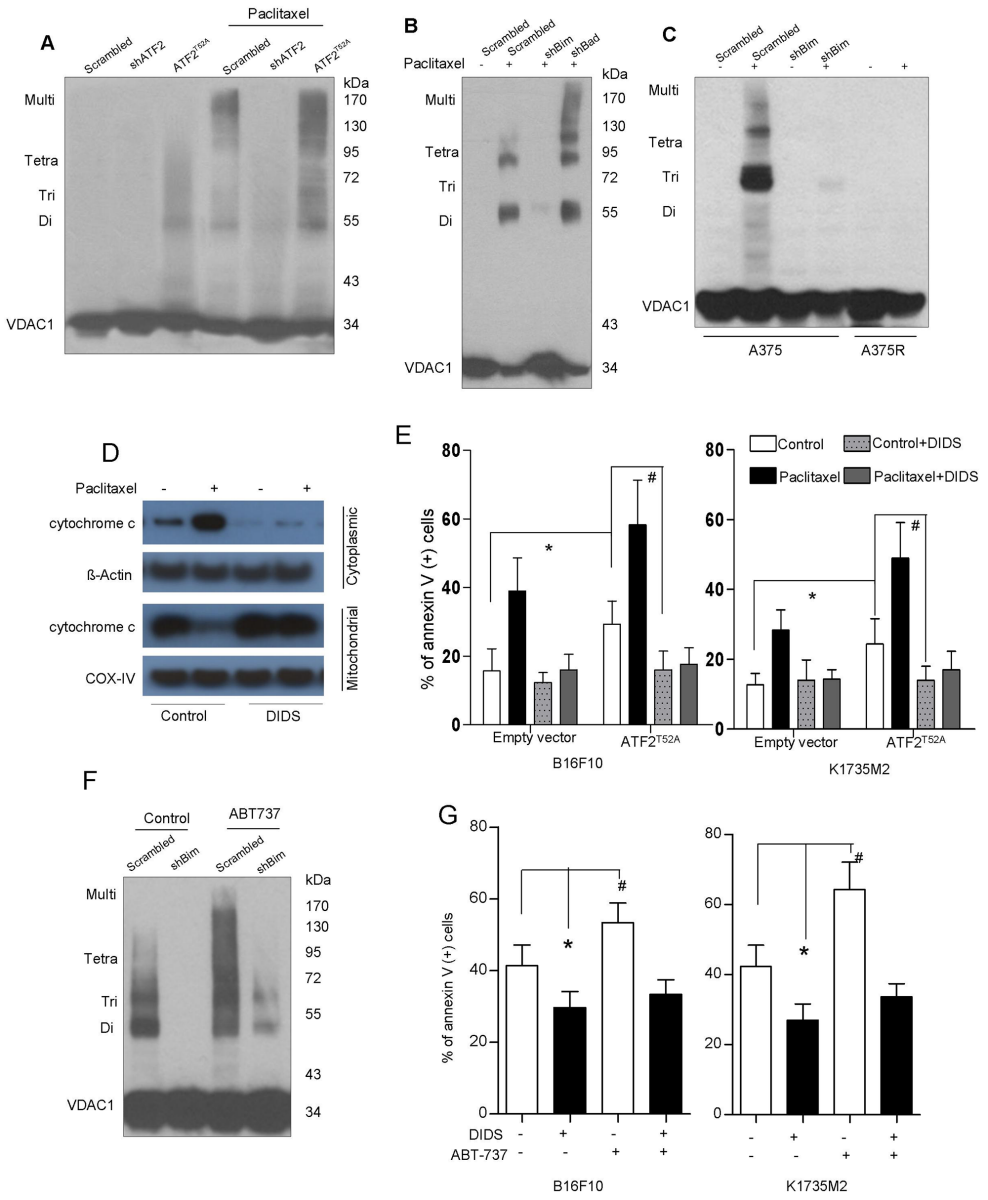
Bim triggers mitochondrial VDAC upregulation and dimerization, Cytochrome c release and apoptosis **A.** B16F10 cells stably transfected with empty vector (EV), ATF2^T52A^, or ATF2 shRNA were treated with paclitaxel (100 nM) for 24 h. The cells were then incubated with EGS (250 μM, 15 min), followed by SDS-PAGE and western blot analysis using an anti-VDAC1 antibody. The positions of the molecular weight protein standards are provided. Representative figures of multiple experiments are shown. **B.** B16F10 cells stably transfected with scrambled or Bim shRNA and Bad shRNA were treated with paclitaxel (100 nM) for 24 h. The cells were then subjected to crosslinking with EGS and western blot analysis using an anti-VDAC1 antibody. **C.** A375 cells stably transfected with scrambled or Bim shRNA and A375R cells were subjected to vemurafenib (5 μM) treatment for 24 h. The cells were then subjected to crosslinking with EGS and western blot analysis using an anti-VDAC1 antibody. **D.** B16F10 cells in the presence or absence of DIDS (100 μM, 1 hour) were treated with paclitaxel (100 nM) for 24 h. Western blot analysis of cytochrome c in the cytosolic and mitochondrial fractions was performed. β-actin and COX-IV were used as loading controls. Representative figures of multiple experiments are shown. **E.** B16F10 cells (left panel) and K1735M2 cells (right panel) stably transfected with empty vector (EV) or ATF2^T52A^ in the presence or absence of DIDS were further treated with paclitaxel (100 nM) for 24 h and subjected to apoptosis analysis using Annexin V/PI staining. Columns represent the mean percentage of annexin V-positive cells from three independent experiments; bars, s.e.m. *, #*p* < 0.01 compared with the corresponding control groups (one-way ANOVA). **F.** A375 cells expressing scrambled or Bim-specific shRNA were treated with ABT737 (1 μM, 24 h) and subjected to crosslinking with EGS and western blot analysis using an anti-VDAC antibody. **G.** B16F10 and K1735M2 cells in the presence or absence of DIDS were treated with ABT737 and subjected to apoptotic cell death analysis using Annexin V/PI staining. Columns represent the mean percentage of annexin V-positive cells from three independent experiments; bars, s.e.m. *p < 0.01, ^#^*p* < 0.01 compared with the control groups (one-way ANOVA).

Previous studies have reported that ATF2 promotes mitochondrial VDAC1 activation and leads to subsequent cytochrome c release [4]. We next determined VDAC activation (the dimerization-oligomerization status of VDAC) in the presence and absence of paclitaxel in B16F10 and K1735M2 cells. As revealed by an EGS-based crosslinking assay, several distinct (68-, 99-, and 136-kDa) protein bands were observed that corresponded to homodimers, trimers, tetramers, and complexes containing VDAC (Supplementary Figure S4A). VDAC oligomerization was dramatically elevated (by as much as 20-fold) upon apoptotic stimuli or in cells expressing cytosolic ATF2 (Figure 4A). In contrast, there was no detectable VDAC homodimers or higher-order oligomerization present in ATF2-depleted cells, even with paclitaxel treatment (Figure 4A), suggesting that upon the induction of apoptosis or cytosolic ATF2 localization, the equilibrium of VDAC monomers and oligomers shifted toward the oligomeric forms of VDAC.

Interestingly, depletion of Bim, but not of Bad, almost completely abolished the formation of oligomeric VDAC (Figure 4B), supporting the hypothesis that Bim but not Bad functions with VDAC1 activation. Similar results were observed in A375 cells (Figure 4C). We further observed that Bim did not alter the total cellular content of VDAC1, as revealed by western blots of proteins not crosslinked with EGS (Supplementary Figure S4B). There was no formation of oligomeric VDAC1 in A375R cells even upon exposure to vemurafenib treatment (Figure 4C), suggesting that VDAC1 is critical for apoptosis in BRAF inhibitor-resistant cells.

ABT-737 was unable to restore the VDAC oligomerization inhibited by Bim depletion in A375 cells (Figure 4F). We also demonstrated that DIDS, a VDAC1 inhibitor, prevented VDAC oligomerization (Supplementary Figure S4C), mitochondrial cytochrome c release (Figure 4D) and apoptosis in B16F10 cells (Figure 4G), even upon expression of ATF2^T52A^ (Figure 4E). Additionally, ABT737 showed only a limited or no ability to overcome the inhibition of apoptosis induced by DIDS (Figure 4G). These results further suggest that Bim promotion by ATF2 facilitates VDAC1 activation for the efflux of cytochrome c and further sensitizes cells to apoptosis.

### ATF2 promotes the dissociation of Bim from Mcl-1

**Figure 5:**
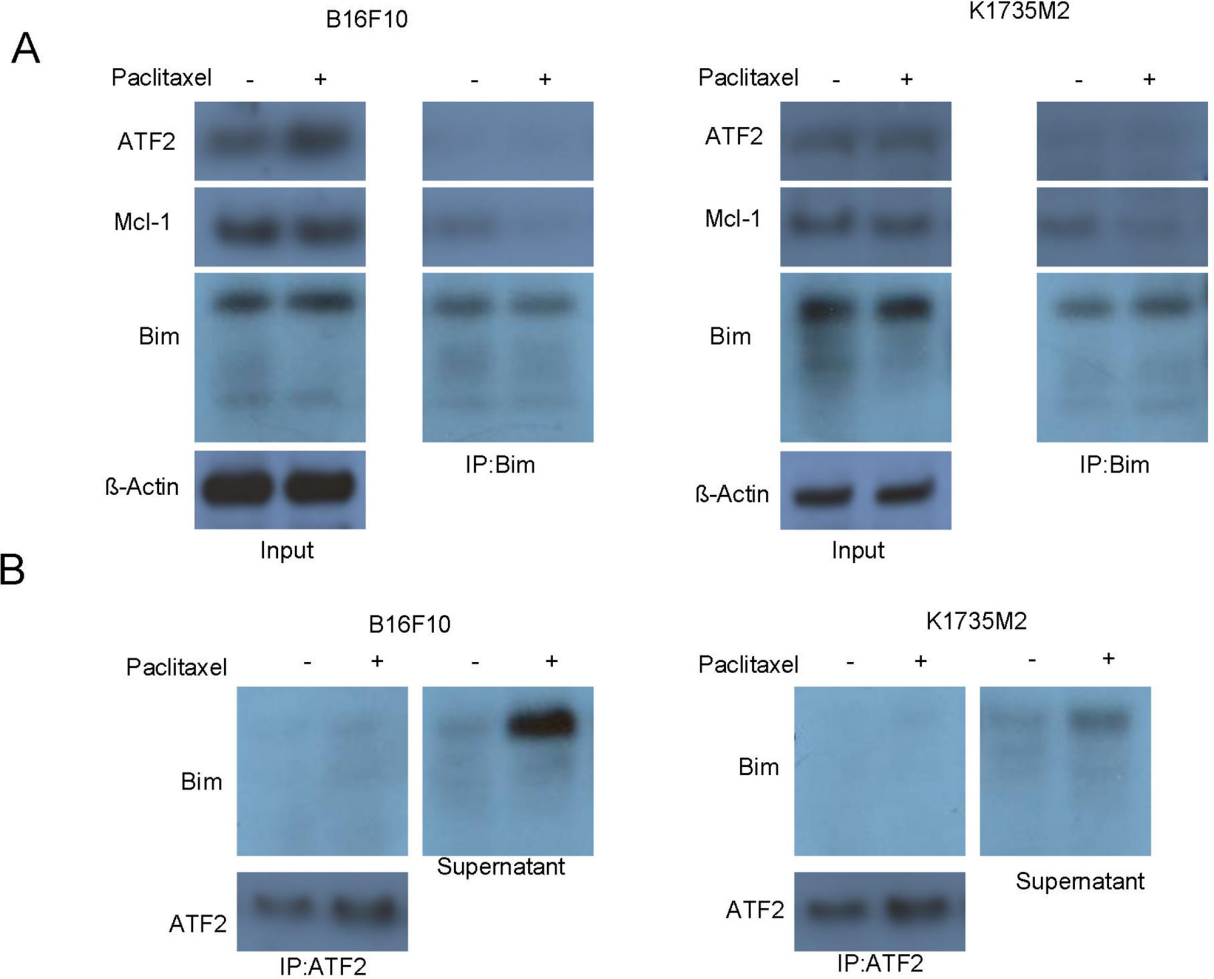
Mitochondrial ATF2 does not directly bind to Bim following paclitaxel treatment **A.** The association of Bim with ATF2 and Mcl-1 was detected by reciprocal immunoprecipitation assays. B16F10 cells (left panel) and K1735M2 cells (right panel) were treated with paclitaxel (100 nM) for 24 h. Bim was immunoprecipitated, and western blot analyses were performed for ATF2, Mcl-1 and Bim. Inputs for coimmunoprecipitation experiments were also subjected to western blot analysis. Representative figures of multiple experiments are shown. **B.** ATF2 was immunoprecipitated from B16F10 cells (left panel) and K1735M2 cells (right panel) treated with paclitaxel. Alternatively, ATF2-depleted supernatants were subjected to western blot analysis to determine the proportion of ATF2-bound and free Bim. Representative figures of multiple experiments are shown.

We next explored whether mitochondrial ATF2 directly binds to Bim to facilitate the efflux of cytochrome c after paclitaxel treatment. First, we immunoprecipitated Bim-containing protein complexes from B16F10 and K1735M2 cells in the presence or absence of paclitaxel and then probed for the indicated proteins by western blot. As shown in Figure 5A, no ATF2 was detected in the precipitated complexes, even following paclitaxel stimulation. Consistent with this, reciprocal immunoprecipitation experiments indicated that Bim was not detected in the ATF2 immunoprecipitated protein complexes (Figure 5B). Some Bim was present in the supernatant, which most likely accounted for paclitaxel-induced mitochondrial apoptosis (Figure 5B) in B16F10 and K1735M2 cells. Together, these results suggest that mitochondrial ATF2 does not directly associate with Bim and most likely activates Bim via indirect mechanisms.

We next addressed whether mitochondrial ATF2 influenced the association or dissociation of Bim with other binding partners. Immunoprecipitation experiments were performed to determine the association profile of Bim in ATF2-expressing B16F10 cells. We found that following paclitaxel treatment, the association of Bim with Mcl-1 was more pronounced compared with Bcl-2 and Bcl-xL in B16F10 cells (Supplementary Figure S5A). The Bim/Mcl-1 complex could be disrupted to a large extent by mitochondrial ATF2 in B16F10 ATF2 ^T52A^-expressing cells (Supplementary Figure S5A, S5B), suggesting that ATF2 facilitated Bim release from the Mcl-1/Bim complex in B16F10 cells. In contrast, Bcl-2 and Bcl-xL remained bound to Bim in these B16F10 cells, even with conditional mitochondrial ATF2 expression (Supplementary Figure S5A).

### ATF2 mitochondrial accumulation promotes the association of Bim and VDAC1

**Figure 6:**
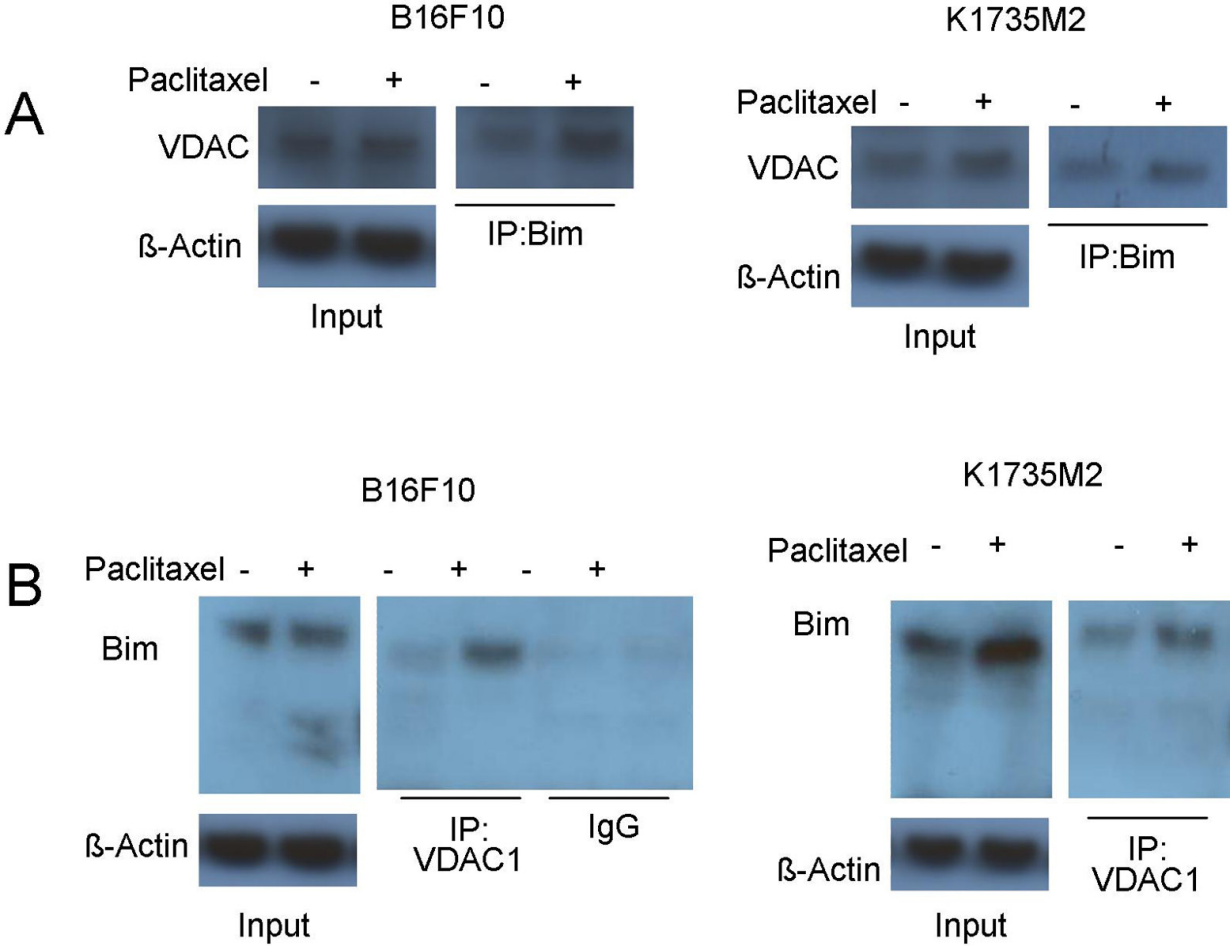
Mitochondrial ATF2 facilitates the association of Bim with VDAC1 following paclitaxel treatment **A.** Bim was immunoprecipitated from paclitaxel-treated (100 nM, 24 h) cells (Left panel: B16F10 cells; Right panel: K1735M2 cells), and western blots were conducted to determine the association of Bim with VDAC1. Representative figures of multiple experiments are shown. **B.** Reciprocal immunoprecipitation-western blot analysis of paclitaxel-treated cells (Left panel: B16F10 cells; Right panel: K1735M2 cells) was performed for VDAC1, followed by western blot for Bim to determine VDAC1-bound Bim. Representative figures of multiple experiments are shown.

The essential role of Bim in mitochondrial ATF2-associated apoptosis and ATF2-dependent VDAC activation raised the possibility that ATF2 might promote the interaction of Bim with VDAC to form mitochondrial permeabilizing pores for cytochrome c release, which have been reported in other systems [30]. First, we addressed whether Bim could directly bind to VDAC1. As revealed by reciprocal immunoprecipitation assays after paclitaxel treatment, the association of Bim with VDAC1 was increased (Figure 6A, 6B) in B16F10 and K1735M2 cells. The level of the Bim-VDAC1 complex detected increased during the course of paclitaxel treatment, and DIDS was able to disrupt this interaction (Supplementary Figure S6A).

In ATF2^T52A^ expressing cells, we found increased accumulation of mitochondrial Bim (Figure 1E), as well as significantly increased levels of VDAC1 protein immunoprecipitated with Bim (Supplementary Figure S5A), suggesting that mitochondrial ATF2 promotes the association of mitochondrial Bim with VDAC1. This is likely derived from the increase in Bim in the mitochondria (Figure 1C), where VDAC1 is localized. The association of other BH3-only proteins with VDAC1 was also examined. As shown in Supplementary Figure S6B, with paclitaxel treatment, no PUMA and a small amount of NOXA bound to VDAC1, indicating that the association of Bim and VDAC1 is specific and physiologically important.

### Bim binds to VDAC1 in a manner that depends on its dissociation from Mcl-1

**Figure 7:**
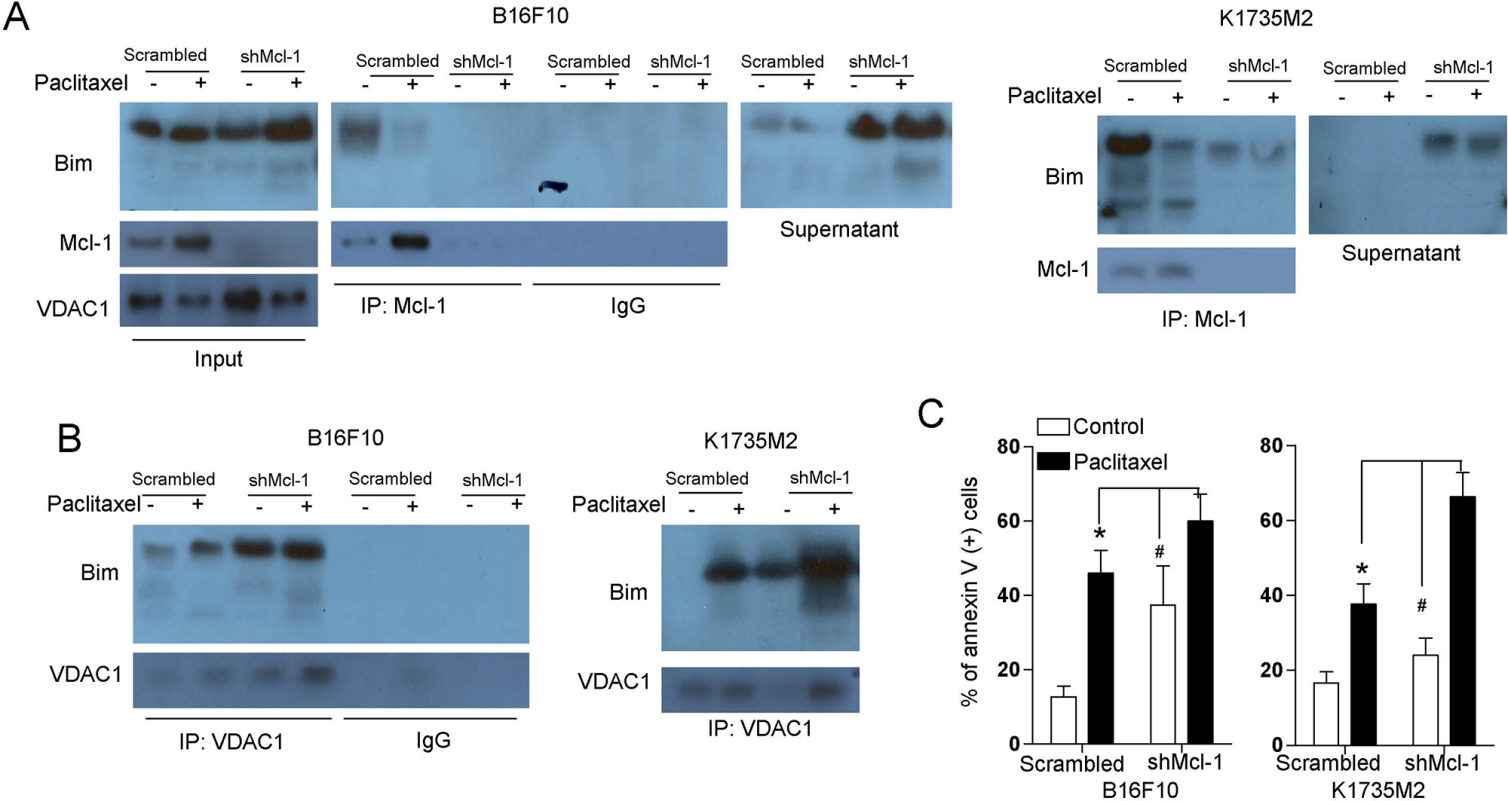
Mcl-1 is involved in the association of Bim with VDAC1 **A.** B16F10 cells (left panel), and K1735M2 cells (right panel) stably transfected with scrambled or Mcl-1 shRNA were treated with paclitaxel (100 nM) for 24 h, and Mcl-1 was immunoprecipitated, followed by immunoblotting for Bim to detect their interaction. Inputs for coimmunoprecipitation experiments were also subjected to western blot analysis. Representative figures of multiple experiments are shown. **B.** B16F10 cells (left panel) and K1735M2 cells (right panel) stably transfected with scrambled or Mcl-1 shRNA were treated with paclitaxel (100 nM) for 24 h, and VDAC1 was immunoprecipitated, followed by immunoblotting for Bim to detect their interaction. Representative figures of multiple experiments are shown. **C.** B16F10 cells (left panel), and K1735M2 cells (right panel) stably transfected with scrambled or Mcl-1 shRNA were treated with paclitaxel (100 nM) for 24 h and then subjected to apoptosis analysis using Annexin V/PI staining. Columns represent the mean percentage of annexin V-positive cells from three independent experiments; bars, s.e.m. *p < 0.01, #*p* < 0.01 compared with the paclitaxel + shMcl-1 group (one-way ANOVA).

Based on the above findings, we next explored the effect of Mcl-1 on the association of VDAC1 and Bim. Our immunoprecipitation analyses indicated that following depletion of Mcl-1, more Bim was released in the supernatant and was not bound to Mcl-1 in B16F10 and K1735M2 cells (Figure 7A). Additionally, Mcl-1 depletion promoted the association of Bim with VDAC1, which was in contrast to the binding of Bim to Mcl-1 (Figure 7B). Moreover, Mcl-1 depletion preceded mitochondrial apoptosis (Figure 7C). Consistently, Mcl-1 depletion-induced Bim/VDAC1 association (Supplementary Figure S7A), cytochrome c release (Supplementary Figure S7B) and mitochondrial cell death (Supplementary Figure S7C) were prevented by DIDS treatment.

To determine whether Bim/VDAC1 association and cell death are a consequence of caspase activation, B16F10 and K1735M2 cells were treated with a pan-caspase inhibitor, Z-VAD-fmk (100 μM), prior to paclitaxel and vemurafenib treatment. We observed that z-VAD-fmk addition prevented caspase activation but not Bim/VDAC1 association and the generation of Bim. Because 100 mM of Z-VAD-fmk was sufficient to block caspase activation, the association of Bim with VDAC1 might be the initial signaling of the mitochondrial apoptotic event rather than a coincident event or a consequence of caspase activation (Supplementary Figure S7D). These results indicate that following paclitaxel treatment in B16F10 cells, the release of Bim from Bim/Mcl-1 complexes promotes VDAC1 and Bim association and then facilitates VDAC1 activation, which is critical for initiating mitochondrial apoptosis.

## DISCUSSION

To the best of our knowledge, the present work provides the first evidence that mitochondrial ATF2 contributes to VDAC1 activation via Bim and Mcl-1, which account for apoptosis induction and BRAF inhibitor resistance in several melanoma cell lines. The present study revealed that various apoptosis-inducing agents, including paclitaxel and vemurafenib, induce mitochondrial localization of ATF2, which sensitizes cells to apoptosis, and was consistent with previous reported results in melanoma [5] and prostate cancer [8], as well as in neurodegenerative diseases, such as Alzheimer's and Parkinson's diseases [31, 32]. Furthermore, we show that ectopic cytosolic expression of ATF2 restored sensitivity to vemurafenib in BRAF inhibitor-resistant melanoma cells, suggesting that BRAF inhibitor-resistant cells indeed maintain their addiction to the ATF2 pathway. We also detected activation of Bim by mitochondrial ATF2. Silencing of Bim in BRAF inhibitor-resistant cells further confirmed that the acquired resistance involves the BH3-only protein Bim [18].

It is well known that Bim is tightly regulated by a variety of transcriptional as well asand post-translational control mechanisms [28, 33]. During endoplasmic reticulum ER(ER) stress, CHOP and C/EBPa cooperate to induce bim transcription by binding to a site within the first intron. In our ATF2-expressing cells, we first found that ATF2-induced Bim upregulation does not occur at the transcriptional level. Bim can be phosphorylated for proteasomal degradation [28, 34, 35], and proteasome-dependent Bim degradation was shown to be critical for the apoptosis of osteoclasts and fibroblasts [36, 37]. We confirmed that although ATF2 cannot directly bind to Bim, ATF2 inhibits Bim proteasomal degradation and promotes its translocation to the mitochondria. This also explains the higher mitochondrial cytochrome c release observed in paclitaxel-treated cells and supports the importance of the regulation of Bim degradation as a priming event to sensitize the apoptotic response. The mechanism by which ATF2 regulates Bim phosphorylation in melanoma remains to be elucidated.

Previous studies demonstrated that following apoptotic stimuli, Bim is released from the Mcl-1, Bcl-xl, and Bcl-w complex to trigger mitochondrial apoptotic signaling events [38, 39, 40]. Here, we revealed that accumulated mitochondrial ATF2 resulting from paclitaxel treatment led to the disassociation of Bim from the Bim/Mcl-1 complex. The observation that immunoprecipitation of Bcl-xL and Bcl-2 family proteins from Bim-containing complexes was not altered by paclitaxel treatment further conforms the specificity of the Bim and Mcl-1 association, which is consistent with the finding that the Bim/Mcl-1 complex is critical for apoptosis modulation in chronic lymphocytic leukemia [41, 42]. Moreover, following ATF2 depletion, Bim was no longer released from the Mcl-1/Bim complex; hence, ATF2 specifically displaced Bim from Mcl-1 and then triggered mitochondrial apoptotic events following apoptotic stress. A similar mechanism was demonstrated in multiple myeloma [43] and HeLa cell apoptosis [44]. In addition, ABT-737, which displaced Bim from Bcl-2, thus functionally mimicking Bim, restored the sensitivity of ATF2-depleted B16F10 and K1735M2 cells to paclitaxel, supporting a role for Bim as a critical apoptotic modulator of mitochondrial ATF2. Considering this observation, we can infer that the displacement of Bim might require coordinated paclitaxel-mediated pro-death signals to neutralize anti-apoptotic potential.

It has been accepted that VDAC1 oligomerization is the general mechanism for MOMP and cytochrome c release from the intermembrane space, leading to apoptosis [45, 46]. In contrast, another controversial report [47] proposed that VDAC1 is not necessary for mitochondrial permeability channels in embryonic fibroblasts. There may be both VDAC1-dependent and VDAC1-independent mechanisms of cytochrome c leakage in different cell types or in response to different apoptosis-inducing treatments [48]. In this study, we obtained direct evidence that VDAC1 oligomerization is essential for apoptotic mitochondrial changes, including cytochrome c release and mitochondrial membrane potential loss in melanoma cells. Moreover, vemurafenib exposure led to VDAC1 oligomerization in the sensitive A375 cell line. In contrast, VDAC1 oligomerization was inhibited in acquired BRAF inhibitor-resistant cells and Bim-depleted A375 cells. Therefore, continued dependence on the Bim/VDAC1 pathway may be an important contributor to BRAF inhibitor-resistant melanoma. Previous reports have suggested that mitochondrial ATF2 may disrupt the HK1-VDAC1 association for apoptosis induction [4]. Consistently, we demonstrated here that the opening of mitochondrial permeability transition pores (loss of mitochondrial membrane potential) during the onset of ATF2-associated apoptosis is inhibited by DIDS, a VDAC1 inhibitor. We also revealed that DIDS significantly inhibits paclitaxel-induced apoptosis. These results support our model that VDAC1 oligomerization plays a critical role in ATF2-dependent apoptogenic cytochrome c release and apoptosis.

To completely understand the detailed mechanism by which ATF2 modulates VDAC1, the elucidation of the molecular structure of VDAC1 and the dynamics of its interaction with other proteins in the presence of ATF2 is essential. Interestingly, we also found that Bim contributes to ATF2-associated apoptosis through VDAC1 oligomerization (activation). In living cells, Bim is localized at microtubules [49, 50], whereas VDAC1 is located in the mitochondria; normally, only a small amount of Bim interacts with VDAC1. In response to apoptotic stimulation as well as to the localization of ATF2 to the mitochondria, Bim translocates to the mitochondria. Indeed, we observed the concomitant association of Bim with VDAC1 in the mitochondria. Consistent with this, Bim has been indicated to directly interact with VDAC in isolated mitochondria and red blood cells. To explore whether caspase activation is initiated by the Bim-VDAC1 association, we evaluated the Bim-VDAC1 association in the presence of z-VAD-fmk, a broad-spectrum pan-caspase inhibitor [51]. We revealed that the association of Bim-VDAC1 was not inhibited by the pan-caspase inhibitor z-VAD-fmk. Therefore, the Bim-VDAC1 association must precede the activation of caspases and may also precede cytochrome c release, which might lead to caspase activation.

Taken together, we showed that mitochondrial ATF2 is involved in the induction of apoptosis and BRAF inhibitor resistance in some melanoma cell lines. We further obtained evidence supporting the novel mechanism that following ATF2 mitochondrial localization, Bim is released from the Bim/Mcl-1 complex and then translocated to the mitochondria. In doing so, mitochondrial ATF2 might function as a critical molecular trigger that drives Bim activation. Moreover, in the mitochondria, Bim activated VDAC1 and resulted in the release of cytochrome c. These findings offer new understanding for the mechanism of ATF2 mitochondrial localization in apoptosis induction and BRAF resistance of melanoma. Furthermore, this new signaling pathway could be a molecular target to develop more effective regimens to induce cell death in melanoma.

## MATERIALS AND METHODS

### Cell culture and reagents

The tumor cell lines B16F10, K1735M2, A375 and M14 (gifts from Prof. Hongbo Luo, Harvard University), as well as derived transfected cells, were used in this study. B16F10 cells were maintained in RPMI 1640, whereas K1735M2, A375 and M14 cells were grown in Dulbecco's modified Eagle's medium; each type of medium was supplemented with 10% fetal bovine serum and 100 units/ml of penicillin G, sodium, and streptomycin sulfate, and all cultures were maintained in a humidified atmosphere of 5% CO2 and 95% air. To generate vemurafenib-resistant cells, continuous selective culture of A375 cells was performed in vemurafenib growth medium with gradually increasing concentrations of vemurafenib from 0.02 to 2.5 μM over 2 months. The cells were maintained in 2.5 μM vemurafenib and designated as A375R. Actinomycin D and cycloheximide were from Sigma-Aldrich (St. Louis, MO, USA). Staurosporine (STS), cisplatin, 4,4′-diisothiocyanostilbene-2,2′-disulfonic acid (DIDS), and propidium iodide were also purchased from Sigma (St. Louis, MO, USA). MitoTracker Red CMXRos and the nuclear export inhibitor leptomycin B (LMB) were purchased from Invitrogen (Eugene, OR, USA) and Santa Cruz Biotechnology (Santa Cruz, CA, USA), respectively. ABT-737 (Selleckchem, Souffelweyersheim, France) was prepared in DMSO at 10 mM. Ethylene glycol-bis (succinimidyl succinate) (EGS) was purchased from Pierce (Rockford, IL). Vemurafenib was obtained from Melone Pharmaceutical Co., Ltd. (Dalian, Liaoning, China), and the stock solution (20 mM) was dissolved in DMSO. Paclitaxel was obtained from Beijing Zhongshuo Pharmaceutical T & D Co., Ltd (Beijing, China) and was dissolved in 100% dimethyl sulfoxide to produce a 1.0 mM stock solution. Z-VAD-fmk (BIOMOL International L.P., PA, USA) was diluted with growth medium to obtain the desired concentrations. RPMI 1640 and DMEM growth media, as well as the supplements, fetal calf serum, L-glutamine, and penicillin-streptomycin, were purchased from Life Technologies (CA, USA).

### Antibodies

The following antibodies were used as primary antibodies: ATF2 (C-19, sc-187), Mcl-1 (S-19, sc-819), cytochrome c (H-104, sc-7159), VDAC1 (FL-283B-6, sc-98708), Bcl-2 (N-19, sc-492), Bim (H-191, sc-11425), PUMAα (H-136, sc-28226), NOXA (FL-103, sc-22764), Bad (C-20, sc-943), Bid (FL-195, sc-11423), Bax (N-20, sc-493), Bok (H-151, sc-11424), Bcl-xL (H-62, sc-7195), β-Actin (H-196, sc-7210) and COX-IV (G-20, sc-69360). All were purchased from Santa Cruz Biotechnology.

### Tetracycline-inducible constructs

For the construction of tetracycline-regulated gene expression vectors expressing ATF2^T52A^ mutants (mitochondria localization), ATF2^T52E^ mutants (nuclear localization), and the WT protein, the vectors, which were a gift from Ze'ev A. Ronai (Sanford-Burnham Medical Research Institute, La Jolla, CA 92037, USA), were amplified by PCR and subcloned into pTHE, resulting in pTHE-WT, ATF2^T52A^, and ATF2^T52E^, which were then transfected into B16F10, K1735M2, A375 and A375R cells with the appropriate blank vector. All PCR-amplified fragments and cloning junctions were verified by DNA sequencing.

### RNA interference

Lentiviral transduction particles for shATF2 (TRCN0000374123), shMcl-1 (TRCN0000273762), shBim (TRCN0000231243), shBad (TRCN0000321208), and a scrambled shRNA (sc-108080) were obtained from Sigma-Aldrich. Cells were transfected at ~70–90% confluence (approximately 1 × 10^5^ cells/ml). The target cells were infected with packaged lentiviral particles in the presence of 10 mg/ml polybrene (Sigma, USA). The gene silencing efficiencies of the shRNAs were evaluated by western blot analysis after 48 h of infection. The appropriate controls were included during the entire shRNA knockdown process to confirm the specificity of the shRNA.

### Cell fractionation and western blot

Cells were harvested by centrifugation, lysed using a protein extraction buffer and homogenized in 250 μl of lysis buffer, as previously described [26]. Subcellular fractions (nuclear, cytosolic and mitochondrial/membrane fractions) were harvested using the subcellular proteome extraction kit (Qiagen, Toronto, ON, Canada). Samples (30 μg protein for each condition) from whole-cell pellets or subcellular fractions were transferred onto PVDF membranes and then incubated with antibodies, following procedures previously described.^20^ Immunoreactive bands were revealed using a 1:5,000 dilution of secondary antibody conjugated to horseradish peroxidase (goat anti-rabbit IgG; sc-2301, Santa Cruz, CA). The blots were reprobed with antibodies against β-actin (Sigma) to ensure equal loading and transfer of proteins. Relative intensities of the bands were evaluated using the Kodak 1D 3.5.4 software (Kodak Scientific Imaging System, Rockville, MD). All critical blots and immunoprecipitation experiments were repeated at least three times. Selected blots were quantified by Image J software.

### Apoptosis assay by annexin V/propidium iodide staining

Apoptosis was measured by staining with fluorescein-conjugated Annexin V and propidium iodide using the annexin V/propidium iodide (PI) detection kit (Biovision) according to the manufacturer's instructions (BD Biosciences, San Jose, CA). A total of 10,000 cells (within whole-cell gates) per replica (3 independent experiments) were subjected to flow cytometry analysis to monitor the green fluorescence of annexin V and the red fluorescence of DNA-bound PI via excitation at 488 nm. The raw data obtained were analyzed with FlowJo software (TreeStar, OR). The results were normalized to the survival of control cells that were treated with DMSO or ethanol.

### Quantitative real-time polymerase chain reaction (qRT-PCR)

The half-life of Bim mRNA was determined by qRT-PCR analysis following actinomycin D treatment. Total cell RNA was isolated using TRIzol according to the manufacturer's instructions (Invitrogen). RNA was then reverse transcribed using the TaqMan Reverse Transcription Reagents Kit (Applied Biosystems) and amplified on a 7300 Real-Time PCR system (Applied Biosystems) with TaqMan gene-specific primers (Applied Biosystems). The relative amount of RNA for each gene was expressed after normalization to β-actin. All standards and samples were tested in triplicate wells, and data were analyzed using SDS software version 2.3.

### Crosslinking experiments for VDAC1

Target cells were washed after the appropriate treatment, and the crosslinking reagent EGS (Pierce, Rockford, IL) was added at a final concentration of 3 mg/ml in PBS (pH 8.3) for 15 min at 30°C. The crosslinker was quenched by the addition of Tris-HCl (pH 7.5) at a final concentration of 20 mM. Samples (50 μg) were then subjected to sodium dodecyl sulfate-polyacrylamide gel electrophoresis (SDS-PAGE) and detected by western blot using an anti-VDAC1 polyclonal antibody. For the EGS control, the quencher was added prior to the crosslinking agents.

### Confocal immunofluorescence assays

Cells from different treatment groups were incubated with MitoTracker Red (25 nM) for 15 min, fixed, permeabilized, and stained with antibodies for the detection of Bim. The primary antibodies were revealed using either goat anti-rabbit or anti-mouse IgG conjugated to Alexa fluor 488 (green) (1:500, diluted in blocking solution) from Molecular Probes-Invitrogen. After 1 hour of incubation, the slides were mounted, and the stained cells were analyzed via fluorescence confocal microscopy (Leica Microsystems Heidelberg GmbH, Heidelberg, Germany), with excitation at 488 nm and emission at 525 nm in 5 non-overlapping fields per experiment. Quantitative colocalization analysis of confocal images was performed using the plugin ‘colocalization threshold’ of WCIF ImageJ software (National Institutes of Health, Bethesda, MD) by at least two independent investigators. The values of Pearson's and Mander's coefficients were calculated using thresholds of the two channels (tM1 and tM2) and reported the overlap of the signals which represents the degree of colocalization.

### Cytochrome c release assay

Isolated tumor cells (5 × 10^7^) were collected and assayed with the Cytochrome c Apoptosis Assay Kit (Cat. #K257–100, Biovision, CA, USA). Briefly, the cells were homogenized with the cytosol extraction buffer provided in the kit and then centrifuged at 10,000 × g for 30 minutes at 4°C; the pellet contained the mitochondrial fraction, and the supernatant was collected as the cytosolic fraction. These fractions were analyzed for cytochrome c by western blot using the cytochrome c antibody provided in the kit.

### Mitochondrial membrane potential detection

The mitochondrial permeability transition was determined by staining the cells with JC-1 (Molecular Probes, Leiden, The Netherlands) as described. Cells were collected by centrifugation and resuspended at a concentration of 1 million cells/ml. Each sample was incubated with JC-1 for 20 min at 37°C and analyzed by flow cytometry, with 10,000 events counted for each sample.

### Immunoprecipitation

Immunoprecipitation analysis was performed using Protein A+G Sepharose Beads (P001-5, Shanghai Yanji Biotechnology) per the manufacturer's instructions. Briefly, protein A+G Sepharose beads were washed and pre-incubated with primary antibody for 1 hour at 4°C. Cells were lysed with CHAPS buffer (20 mM Tris-HCl, pH 7.5; 150 mM NaCl; 1 mM EDTA; 2% CHAPS; Calbiochem). The beads were incubated in the presence of 800 μg of protein overnight with constant agitation at 4°C. Bound protein was eluted off the beads by boiling in sample buffer and was then analyzed by western blot.

### Statistical analysis

Statistics for the data were calculated with GraphPad Prism version 4 software (GraphPad Software, Inc., La Jolla, CA, USA). Quantitative analysis results are represented as histograms, and mean values and error bars were calculated from three independent experiments. Statistical comparisons between groups were performed by 2-way ANOVA.

## SUPPLEMENTARY FIGURES


