# Supplementary material for: Mitochondrial ATF2 translocation contributes to apoptosis induction and BRAF inhibitor resistance in melanoma through the interaction of Bim with VDAC1
Source: Oncotarget. 2015 Oct 9;6(34):36338–53. doi: 10.18632/oncotarget.5537 (PMC4742181; doi:10.18632/oncotarget.5537)
Supplement: Supplementary file 1 [file oncotarget-06-36338-s001.pdf]

SUPPLEMENTARY FIGURES

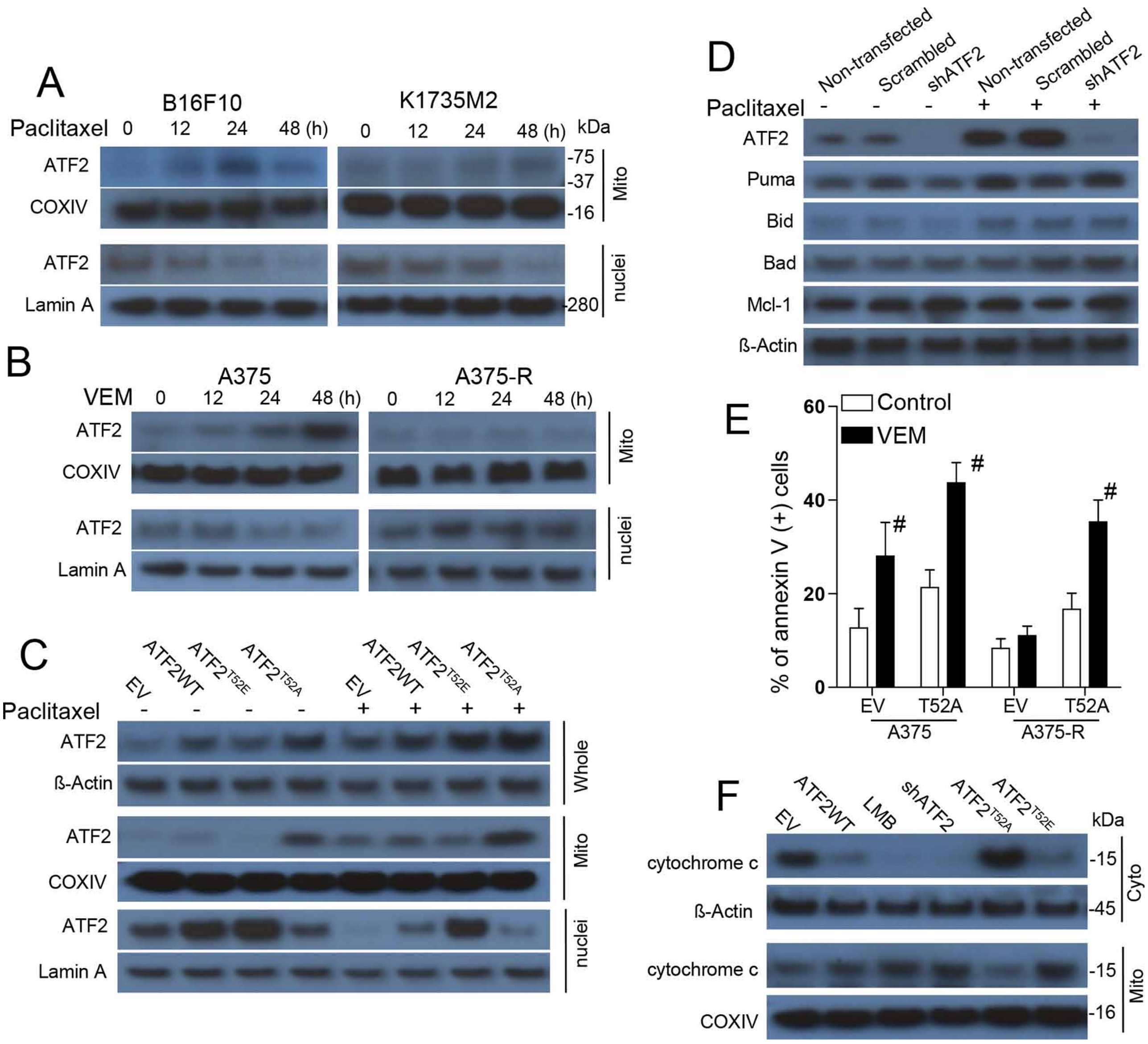

Supplementary Figure S1: Mitochondrial Localization of ATF2 following paclitaxel and vemurafenib stress was related to cytochrome c release and apoptosis. B16F10 and K1735M2 cells were treated with paclitaxel (100 nM) for the indicated times **A.** A375 and A375R cells were treated with 5 µM vemurafenib (VEM) for the indicated times **B.** Western blot analysis were performed for mitochondrial and nuclei ATF2. Cox-IV, Lamin A were probed as a loading control. Representative figures of multiple experiments are shown. **C.** B16F10 cells transfected as indicated were subjected to incubation with paclitaxel (100 nM, 24 h). Whole, nuclei and mitochondrial fractions were subjected to western blotting with ATF2. COXIV, Lamin A and β-Actin were probed as the loading control. Representative figures of multiple experiments are shown. **D.** Scrambled or shATF2-infected B16F10 cells, treated with paclitaxel (100 nM, 24 h), were subject to western blot analysis on indicated Bcl-2 family proteins. Representative figures of multiple experiments are shown. **E.** A375 and A375R cells, transfected with empty vector (EV) or ATF2T52A, were further stressed with vemurafenib (5 µM) for 24 hrs and subjected to apoptosis measurement by Annexin V/PI staining. Columns represent the mean percentage of annexin V-positive cells from three independent experiments; bars, s.e.m. # $P < 0.01$, compared with the corresponding control groups (one-way ANOVA). **F.** B16F10 cells were transfected with empty vector (EV), ATF2 (WT), ATF2^{T52A}, or ATF2^{T52E}, as well as ATF2 shRNA and then treated with paclitaxel (100 nM, 24 h). Leptomycin B (LMB) (40 ng/ml, 6 h) was pre-incubated with B16F10 cells to prevent mitochondrial accumulation of ATF2. Cytosolic and mitochondrial fractions were subjected to western blotting with cytochrome c. β-actin and COX-IV was used as loading control.

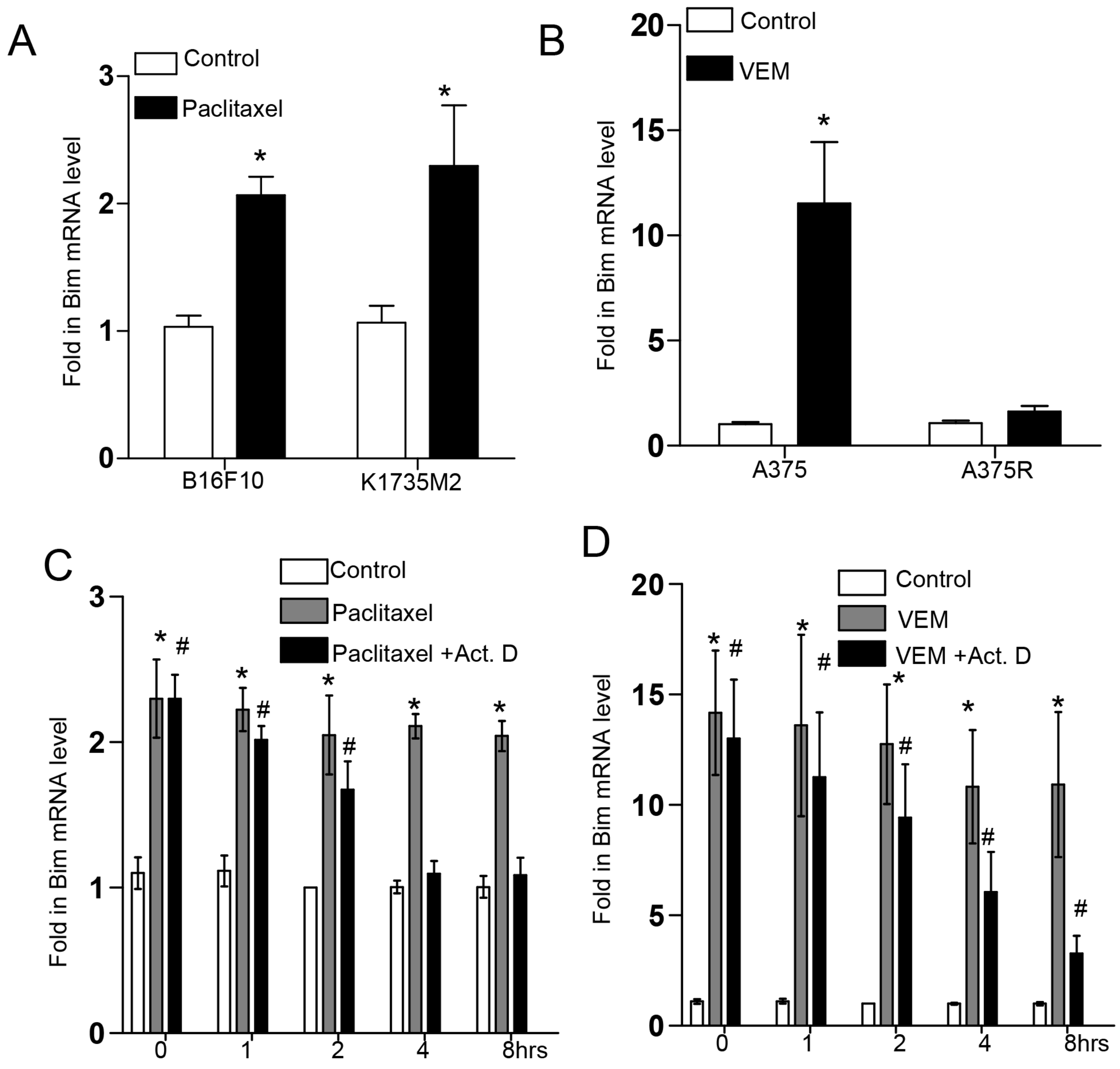

Supplementary Figure S2: Bim is involved in apoptotic stress and BRAF resistance. Analyses of Bim mRNA levels in B16F10 and K1735M2 cells treated with paclitaxel for 24 hrs **A.** and A375 and A375R cells treated with 5 μM vemurafenib (VEM) **B.** by quantitative real-time PCR. RNA expression is expressed relative to the levels detected in the 2-h untreated sample (assigned *a* value of 1). bars, s.e.m. At least three independent experiments were performed. *$p < 0.01$ compared with the corresponding control (one-way ANOVA). Bim expression in B16F10 cells **C.** and A375 cells **D.** pretreated with the transcription inhibitor actinomycin D (ActD, 2.5 μg/ml) by a time course as indicated, followed of paclitaxel and vemurafenib treatment for 24 h, respectively. RNA expression is expressed relative to the levels detected in the 2-h untreated sample (assigned *a* value of 1). bars, s.e.m. At least three independent experiments were performed. *$p < 0.01$, #$p < 0.01$ compared with the corresponding control (one-way ANOVA).

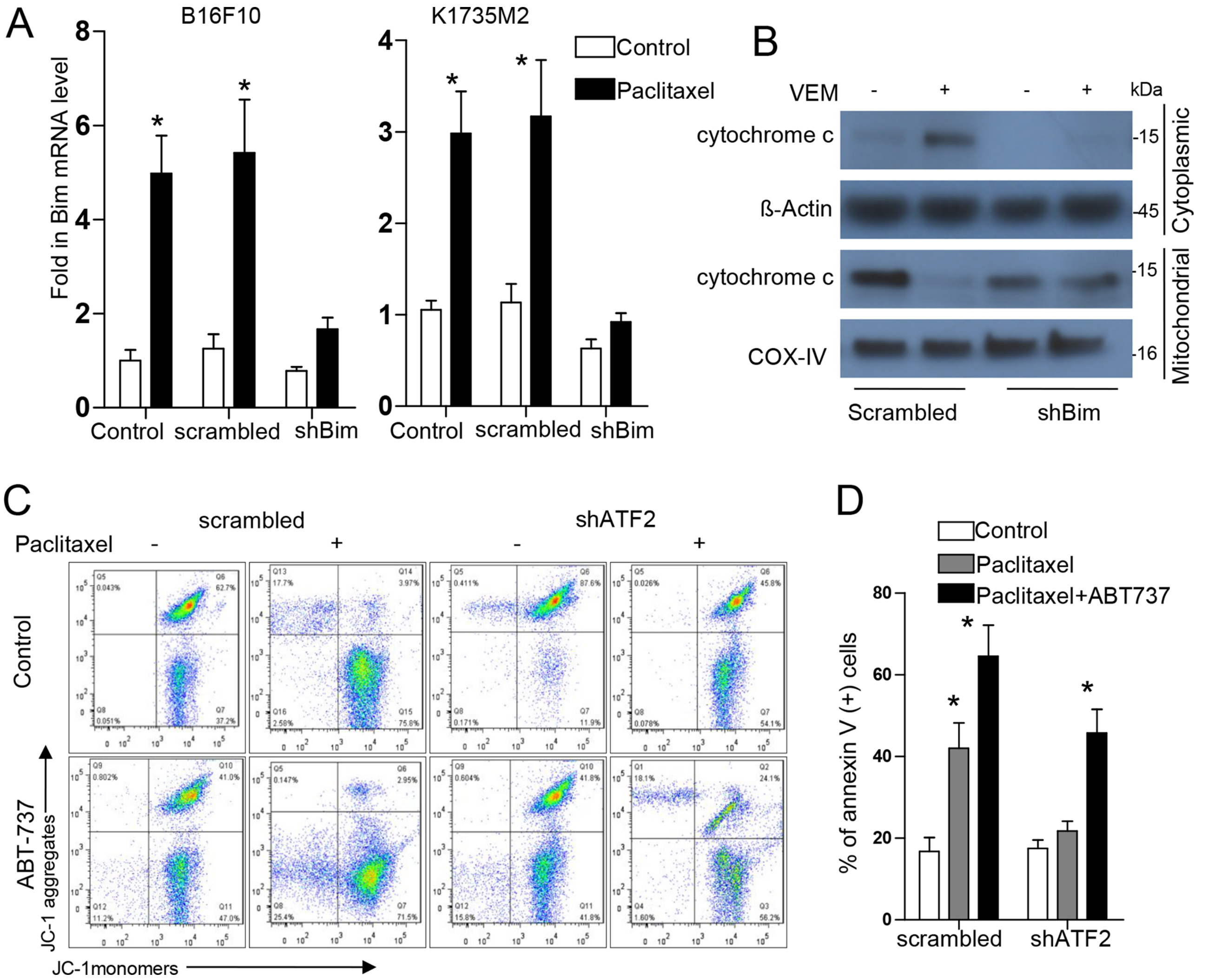

Supplementary Figure S3: ABT-737 sensitized the ATF2-depleted cells in mitochondrial membrane potential and apoptosis during apoptostic stress. A. B16F10, K1735M2 cells, transfected with empty vector (EV), shBim, were subjected to paclitaxel stress with higher dosage of 150 nM and longer duration for 72 hours. Bim RNA expression is measured using real time qRT-PCR and expressed relative to the levels detected in the 2-h untreated sample (assigned *a* value of 1). bars, s.e.m. At least three independent experiments were performed. *$p < 0.01$ compared with the corresponding control (one-way ANOVA). **B.** A375 cells expressing scrambled or Bim shRNA were treated with 5 µM vemurafenib (VEM) and subjected to western blotting with cytosolic and mitochondrial cytochrome c. β-actin and COX-IV was used as loading control. Representative figures of multiple experiments are shown. **C.** B16F10 Cells, transfected by scrambled or ATF2-specific shRNA, were exposed in the absence or presence of ABT737 and treated with paclitaxel, then were subjected to JC-1 flow cytometry analysis. The data are representatives from three independent experiments. **D.** B16F10 Cells, transfected by scrambled or ATF2-specific shRNA, were exposed in the absence or presence of ABT737 and followed with paclitaxel stress. The cells were then subjected to measurement of apoptosis by Annexin V/PI staining. Columns represent the mean percentage of annexin V-positive cells from three independent experiments; bars, s.e.m. *$p < 0.01$ compared with the corresponding control (one-way ANOVA).

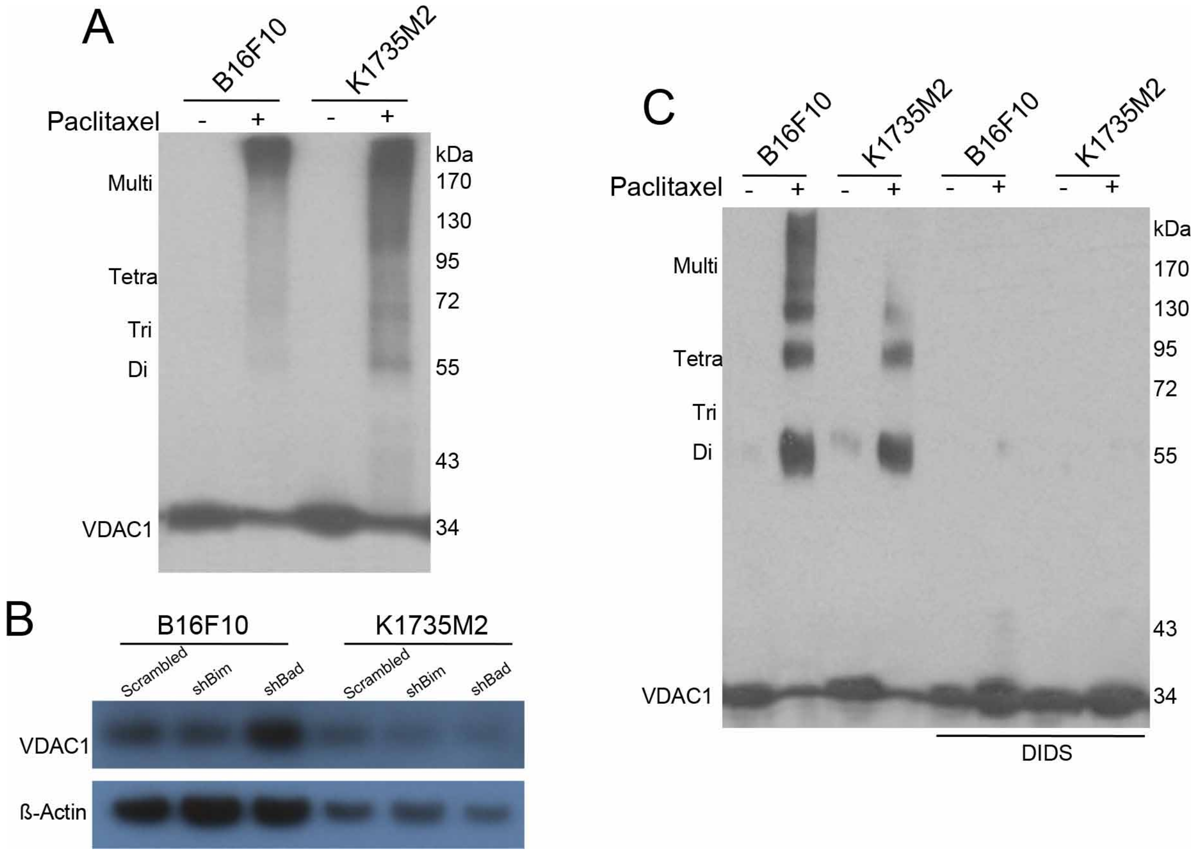

Supplementary Figure S4: Apoptosis stimuli induced VDAC oligomerization was inhibited by DIDS. A. B16F10, K1735M2 cells were treated with paclitaxel (100 nM; 24 h) and subjected to cross-linking with EGS (250 μM, 15 min) and immunoblotting using anti-VDAC antibodies. The positions of molecular size protein standards are provided. Representative figures of multiple experiments are shown. **B.** B16F10, K1735M2 cells, transfected by scrambled or Bim-specific shRNA and Bad-specific shRNA, were subjected to whole-cell lysis western blot analysis for VDAC1. β-Actin probed as a loading control. Representative figures of multiple experiments are shown. **C.** B16F10, K1735M2 cells were incubated in the absence and presence of DIDS (100 μM, 1 h), followed with paclitaxel stress. The cells were then subjected to cross-linking with EGS (250 μM) and immunoblotting using anti-VDAC antibody. The positions of molecular size protein standards are provided. Representative figures of multiple experiments are shown.

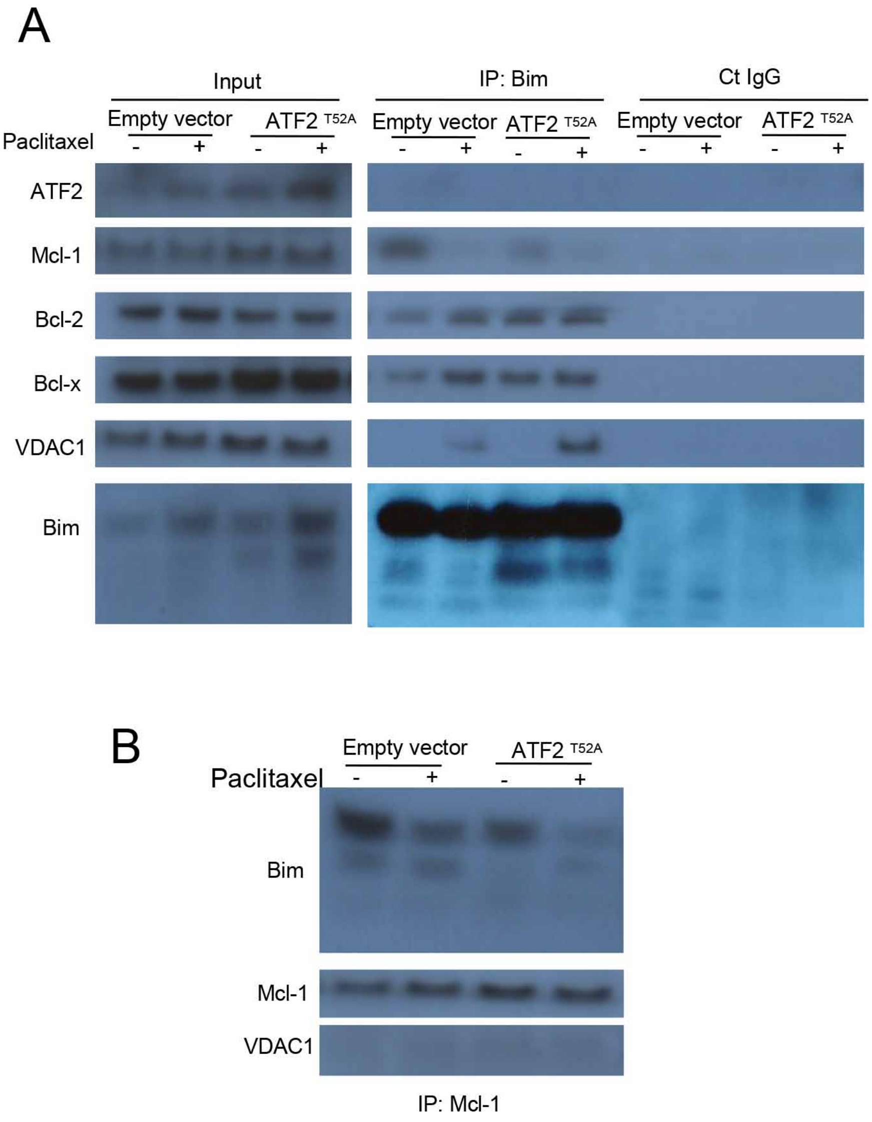

Supplementary Figure S5: Mitochondria ATF2 triggers the release of Bim from Mcl-1 following paclitaxel stress. **A.** B16F10 cells, transfected with EV, $ATF2^{T52A}$, were treated with paclitaxel (100 nM) for 24 hors. Bim was immunoprecipitated and immunoblot analyses were performed for ATF2, Mcl-1, Bcl-2, Bcl-xL, VDAC1, and Bim. Inputs for coimmunoprecipitations were also subjected to immunoblot analysis. Representative figures of multiple experiments are shown. **B.** B16F10 cells, transfected with EV, ATF2T52A, were treated with paclitaxel (100 nM). Mcl-1 was immunoprecipitated and immunoblot analyses were performed for Bim, VDAC1, Mcl-1. Representative figures of multiple experiments are shown.

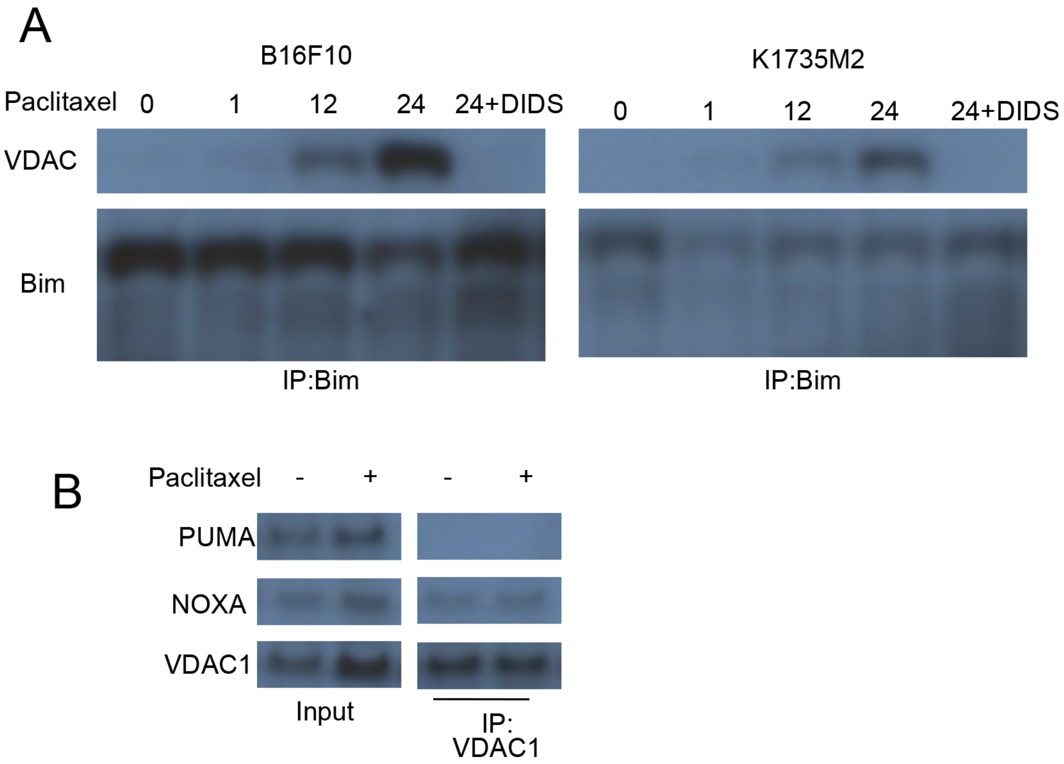

Supplementary Figure S6: Apoptotic stress inducted assiciation of VDAC1 with Bim, instead of PUMA, NOXA. A. Bim was immunoprecipitated from paclitaxel (100 nM)-treated cells for the indicated time couse followed by immunoblotting to determine VDAC1 association. To prevent the VDAC1 and Bim assiciation, DIDS (100 µM, 1 h) was incubated and followed with paclitaxel stress for 24 hrs. Representative figures of multiple experiments are shown. **B.** VDAC1 was immunoprecipitated from paclitaxel-treated cells followed by immunoblotting to determine whether PUMA and NOXA could bind to VDAC. Inputs for coimmunoprecipitations were also subjected to immunoblot analysis. Representative figures of multiple experiments are shown.

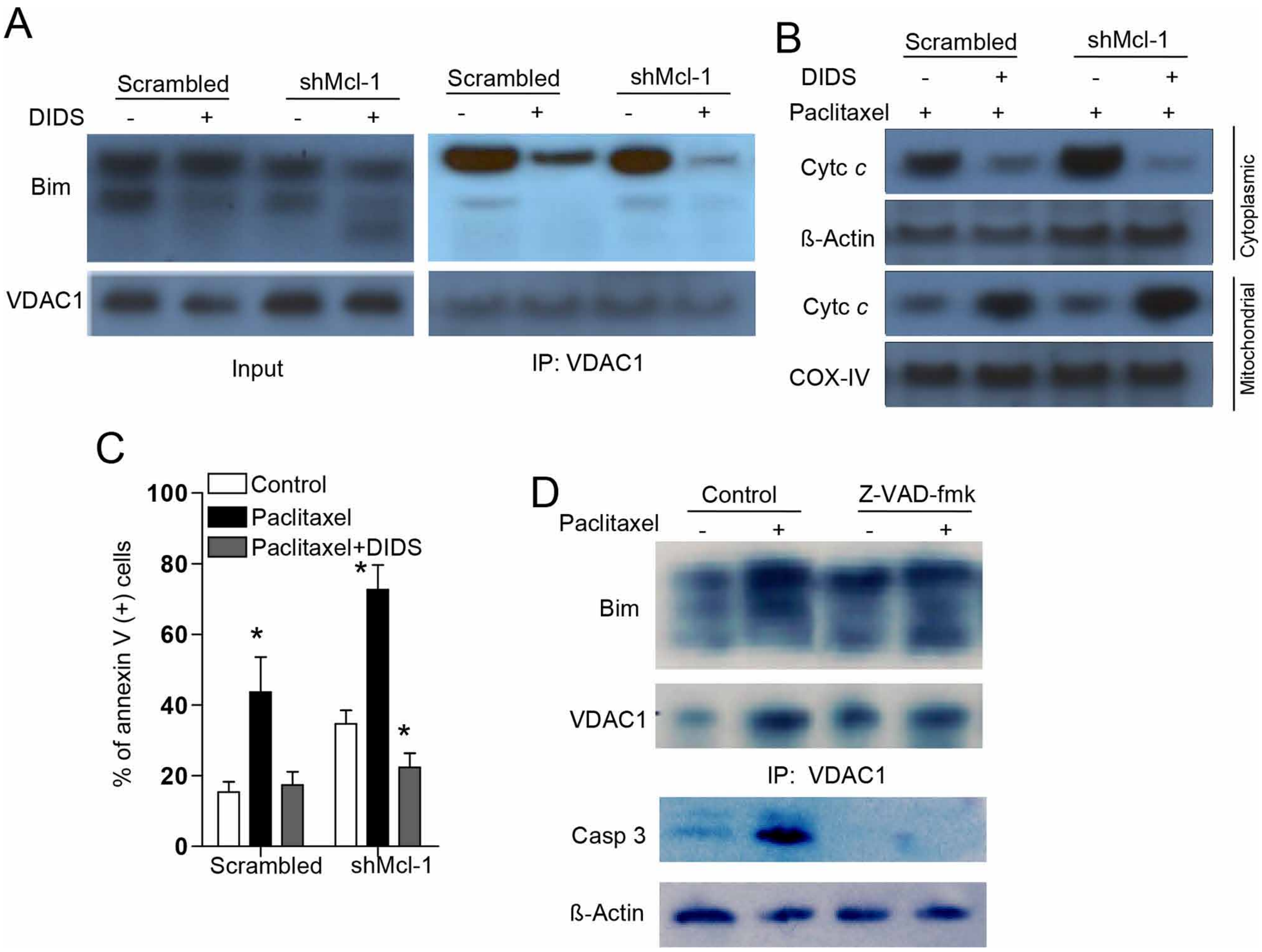

Supplementary Figure S7: VDAC1 inhibitor, DIDS prevented association of Bim/VDAC1, cytc c release, and cell death in B16F10 during Mcl-1 depletion. A. B16F10 cells, transfected with scrambled shRNA, shMcl-1, were incubated with DIDS (100 µM; 1 h), followed by paclitaxel stress(100 nM) for 12 h. VDAC1 was immunoprecipitated and immunoblot analyses were performed for Bim. Inputs for coimmunoprecipitations were also subjected to immunoblot analysis. **B.** B16F10 cells, transfected with scrambled shRNA, shMcl-1, were incubated with DIDS, followed by paclitaxel stress(100 nM) for 12 h. Cytosolic and mitochondrial fractions were subjected to cytochrome *c* (Cytc c) immunoblot. β-actin and COX-IV was used as loading control for the cytosolic and Mito fractions, respectively. **C.** B16F10 cells, transfected with scrambled shRNA, shMcl-1, were incubated with DIDS, followed by paclitaxel stress(100 nM) for 12 h and were subjected to apoptosis measurement by Annexin V/PI staining. Columns represent the mean percentage of annexin V-positive cells from three independent experiments; bars, s.e.m. *$P < 0.01$, compared with the corresponding control groups (one-way ANOVA). **D.** B16F10 cells were incubated with zVAD-fmk (100 µM) for 1 h prior to Paclitaxel treatment. VDAC1 was immunoprecipitated and immunoblot analyses were performed for Bim and expression of caspase-3 was determined by immunoblot analyses.
